# Use of diaphragm thickening fraction combined with rapid shallow breathing index for predicting success of weaning from mechanical ventilator in medical patients

**DOI:** 10.1186/s40560-018-0277-9

**Published:** 2018-02-02

**Authors:** Pattarin Pirompanich, Sasithon Romsaiyut

**Affiliations:** Division of Pulmonary and Critical Care Medicine, Department of Medicine, Faculty of Medicine, Thammasat University, Pathumthani, 12120 Thailand

**Keywords:** Diaphragm, Diaphragm thickening fraction, Ultrasonography, Weaning, Rapid shallow breathing index

## Abstract

**Background:**

Weaning failure is a crucial hindrance in critically ill patients. Rapid shallow breathing index (RSBI), a well-known weaning index, has some limitations in predicting weaning outcomes. A new weaning index using point-of-care ultrasound with diaphragmic thickening fraction (DTF) has potential benefits for improving weaning success. The aim of this study was to evaluate the efficacy of a combination of DTF and RSBI for predicting successful weaning compared to RSBI alone.

**Methods:**

This prospective study enrolled patients from the medical intensive care unit or ward who were using mechanical ventilation and readied for weaning. Patients underwent a spontaneous breathing trial (SBT) for 1 h, and then, both hemi-diaphragms were visualized in the zone of apposition using a 10-MHz linear probe. Diaphragm thickness was recorded at the end of inspiration and expiration which supposed the lung volume equal to total lung capacity (TLC) and residual volume (RV), respectively, and the DTF was calculated as a percentage from this formula: thickness at TLC minus thickness at RV divided by thickness at RV. In addition, RSBI was calculated at 1 min after SBT. Weaning failure was defined as the inability to maintain spontaneous breathing within 48 h.

**Results:**

Of the 34 patients enrolled, the mean (± SD) age was 66.5 (± 13.5) years. There were 25 patients with weaning success, 9 patients in the weaning failure group. The receiver operating characteristic curves of right and left DTF and the RSBI for the prediction of successful weaning were 0.951, 0.700, and 0.709, respectively. The most accurate cutoff value for prediction of successful weaning was right DTF ≥ 26% (sensitivity of 96%, specificity of 68%, positive predictive value of 89%, negative predictive value of 86%). The combination of right DTF ≥ 26% and RSBI ≤ 105 increased specificity to 78% but slightly decreased sensitivity to 92%. Intra-observer correlation increased sharply to almost 0.9 in the first ten patients and slightly increased after that.

**Conclusions:**

Point-of-care ultrasound to assess diaphragm function has an excellent learning curve and helps physicians determine weaning readiness in critically ill patients. The combination of right DTF and RSBI greatly improved the accuracy for prediction of successful weaning compared to RSBI alone.

**Trial registration:**

Thai Clinical Trials Registry, TCTR20171025001. Retrospectively registered on October 23, 2017.

## Background

The diaphragm, a major muscle of inspiration, plays a crucial role in the pathophysiology of respiratory failure [1]. A study from Wait et al. of the diaphragmatic thickness-lung volume relationship in vivo concluded that diaphragmatic thickening fraction (DTF) is directly related to lung volume and may be a useful technique for evaluating diaphragmatic function [2].

Difficult weaning is when patients cannot wean from mechanical ventilation within 7 days or require up to three spontaneous breathing trials (SBT) [3]; the prevalence of difficult weaning is about 20% in patients requiring mechanical ventilation [4]. Patients who require mechanical ventilation for longer durations have higher rates of mortality [4] and morbidity, including ventilator-associated pneumonia [5]; consequently, mechanical ventilation should be discontinued as soon as patients are capable of breathing [6]. Discontinuing mechanical ventilation in a timely and safe way is, therefore, a priority, and strategies that assist in discontinuation should be robustly evaluated [7].

Currently, there are many weaning parameters such as minute ventilation [8], maximum inspiratory pressure (MIP) [9], rapid shallow breathing index (RSBI: the ratio of respiratory frequency to tidal volume) [8], tracheal airway occlusion pressure 0.1 s (P 0.1) [10], and CROP index (dynamic compliance, respiratory rate, oxygenation, maximum inspiratory pressure index) [11]. Among those, the most accurate weaning parameter is RSBI [8]. An RSBI of less than 105 can predict weaning success from mechanical ventilation with sensitivity, specificity, positive predictive value (PPV), and negative predictive value (NPV) of 0.97, 0.64, 0.78, and 0.95, respectively [8]. Nonetheless, its low specificity and PPV can still lead to errors in weaning assessment. In this study, we aim to find a new method for improving accuracy and precision of weaning parameter.

From previous studies [12–14], the common etiology of difficult weaning has been diaphragmatic dysfunction, which progressively worsens while using mechanical ventilation [15]. Diaphragm function evaluation techniques, such as fluoroscopy [16], phrenic nerve stimulation [17, 18], dynamic magnetic resonance imaging of the diaphragm [19], and trans-diaphragmatic pressure measurement [20], have limitations: ionizing radiation exposure, low availability, invasiveness, and need for patient transportation. In contrast to those methods, ultrasound is safe, non-invasive, without radiation hazards, and available at the bedside. Diaphragm function can be evaluated by point-of-care ultrasound, including diaphragmatic excursion [8, 11, 13, 21], diaphragmatic thickness, and DTF [22, 23]. Using as a weaning parameter, a right DTF of more than 36% in semi-upright position was associated with weaning success but not better than RSBI [22]. The aim of our study was to assess the effectiveness of DTF combined with RSBI compared to RSBI alone for the predicted success rate of weaning.

## Methods

This prospective study was performed between April and December 2016 in a tertiary care teaching hospital. Patients were enrolled to this study from medical wards or the medical intensive care unit if they were ready for weaning from mechanical ventilation and could tolerate the SBT for 60 min. Approval was obtained from the Ethics Committee of the Faculty of Medicine, Thammasat University, Thailand (IRB No. MTU-EC-IM-1-048/59), and the study was conducted according to the Declaration of Helsinki. All patients or relatives of the patients gave written informed consent prior to participation. The co-author (SR), a pulmonology fellow, worked with a principle investigator (PP), an intensivist, who was well trained in point-of-care ultrasound, with a specific interest in thoracic ultrasound.

### Patient selection

Inclusion criteria were age ≥ 18 years, respiratory failure with mechanical ventilation more than 24 h, and the ability to tolerate SBT for 1 h before ultrasound. Patient readiness for weaning must have met all criteria: using fraction of inspired oxygen (F_I_O_2_) < 0.5 and PEEP ≤ 5 cmH_2_O, PaO_2_/F_I_O_2_ > 200, respiratory rate ≤ 30 breaths/min, stable hemodynamic (absence or low-dose vasopressors required), good consciousness and cooperative, minimal secretion, and effectiveness of cough. Exclusion criteria were neuromuscular diseases or diaphragmatic paralysis and tracheostomized patients.

### Study design

The patients who met the inclusion criteria above were disconnected from the ventilator to SBT for 1 h using T-piece with oxygen supplementation (F_I_O_2_ of 0.21–0.5) to achieve pulse oxygen saturation (SpO_2_) ≥ 94%. RSBI, MIP, and maximum expiratory pressure (MEP) were recorded after SBT for 1 min by attending physicians who were not involved in this study. MIP was measured by the same pressure gauge in all patients in a semi-upright position. Each participant was asked to perform three MIP maneuvers; the highest negative MIP was recorded. Then, sitting in a semi-recumbent position, both diaphragms were evaluated by B-mode and M-mode ultrasound subcostal views to exclude paradoxical movement [24]. After that, diaphragm ultrasound scans on both sides were performed. Attending physicians were not allowed to know the results of ultrasound measurements.

Weaning failure was defined by the inability to maintain spontaneous breathing for at least 48 h, without any ventilator support [25]. Criteria regarding the inability for the spontaneous breathing were change in mental status, respiratory rate > 35 breaths/min, hemodynamic instability (heart rate > 140/min, systolic blood pressure > 180 or < 90 mmHg), and signs of increased work of breathing. In contrast, weaning success was defined by the ability to maintain spontaneous breathing for at least 48 h, without any ventilator support and without criteria for failure of spontaneous breath.

### Diaphragm ultrasound

Ultrasound was performed using 10-MHz linear probe from LOGIQ C5 Premium (GE Medical System, China). The patients were in a semi-recumbent position. The transducer was placed vertical to the chest wall, at the eighth or ninth intercostal space, between the anterior axillary and the mid-axillary lines, to observe the zone of apposition of the muscle 0.5 to 2 cm below the costophrenic sinus [26]. The inferior border of the costophrenic sinus can be demonstrated by the level of lung artifact caused by the ultrasound reflected by the air in the lung.

**Fig. 1:**
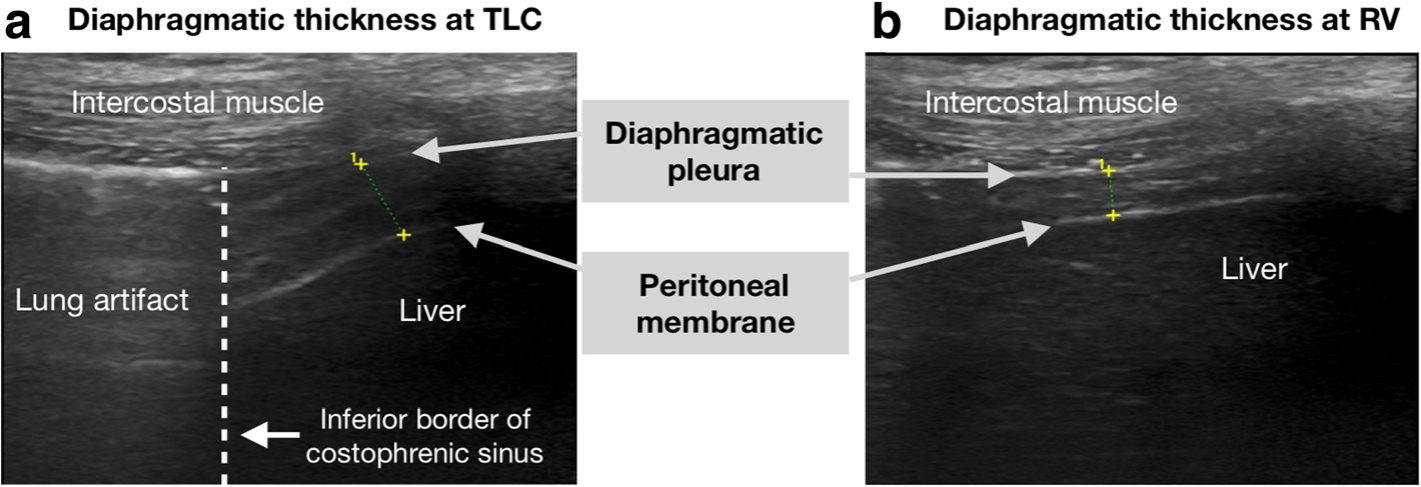
Ultrasound diaphragm shows three layers of diaphragm and diaphragmatic thickness at total lung capacity (TLC) in (**a**) and residual volume (RV) in (**b**). The inferior border of the costophrenic sinus can be demonstrated clearly in (**a**) by the level of lung artifact caused by the ultrasound reflected by the air in the lung

The diaphragm structure has three layers (Fig. 1): two parallel echoic lines (the diaphragmatic pleura and the peritoneal membrane) and a hypoechoic structure between them (the muscle). The patients were asked to deeply inhale to total lung capacity (TLC) and then fully exhale to residual volume (RV). Diaphragm images, first on the right, were captured, stored, and measured for diaphragm thickness in maximum thickening at TLC and minimum thickening at RV in the same breath. On B-mode, the diaphragm thickness was measured from the middle of the pleural line to the middle of the peritoneal line. Then, the DTF percentage was calculated of each diaphragm from the following formula: thickness at the end of inspiration minus thickness at the end of expiration, divided by thickness at the end of expiration, then multiplied by 100 [2]. We measured the DTF of three breaths on each side and used the mean value for analysis.

### Statistical analysis

Data are presented as mean (± SD), median (IQR, interquartile range), and proportion (%) when appropriate. Fisher’s exact test, when necessary, was used for comparisons among categorical variables. *T* test and Mann–Whitney *U* test were utilized for continuous data. For inter-observer variability, 15 patients were analyzed by two different operators. Intra-observer variability was assessed measuring diaphragm thickness twice consecutively. Diagnostic test was used to calculate sensitivity, specificity, positive predictive value, and negative predictive value of DTF in weaning parameters. Pearson correlation evaluated the relationship between DTF and MIP. Receiver operating characteristic (ROC) curve analysis was performed to assess diaphragm DTF, RSBI, and combined RSBI and DTF ability to predict patients who would succeed at weaning or fail. The Spearman coefficient was used to evaluate correlations. A two-tailed *p* value of less than 0.05 was taken to indicate statistical significance.

## Results

**Fig. 2:**
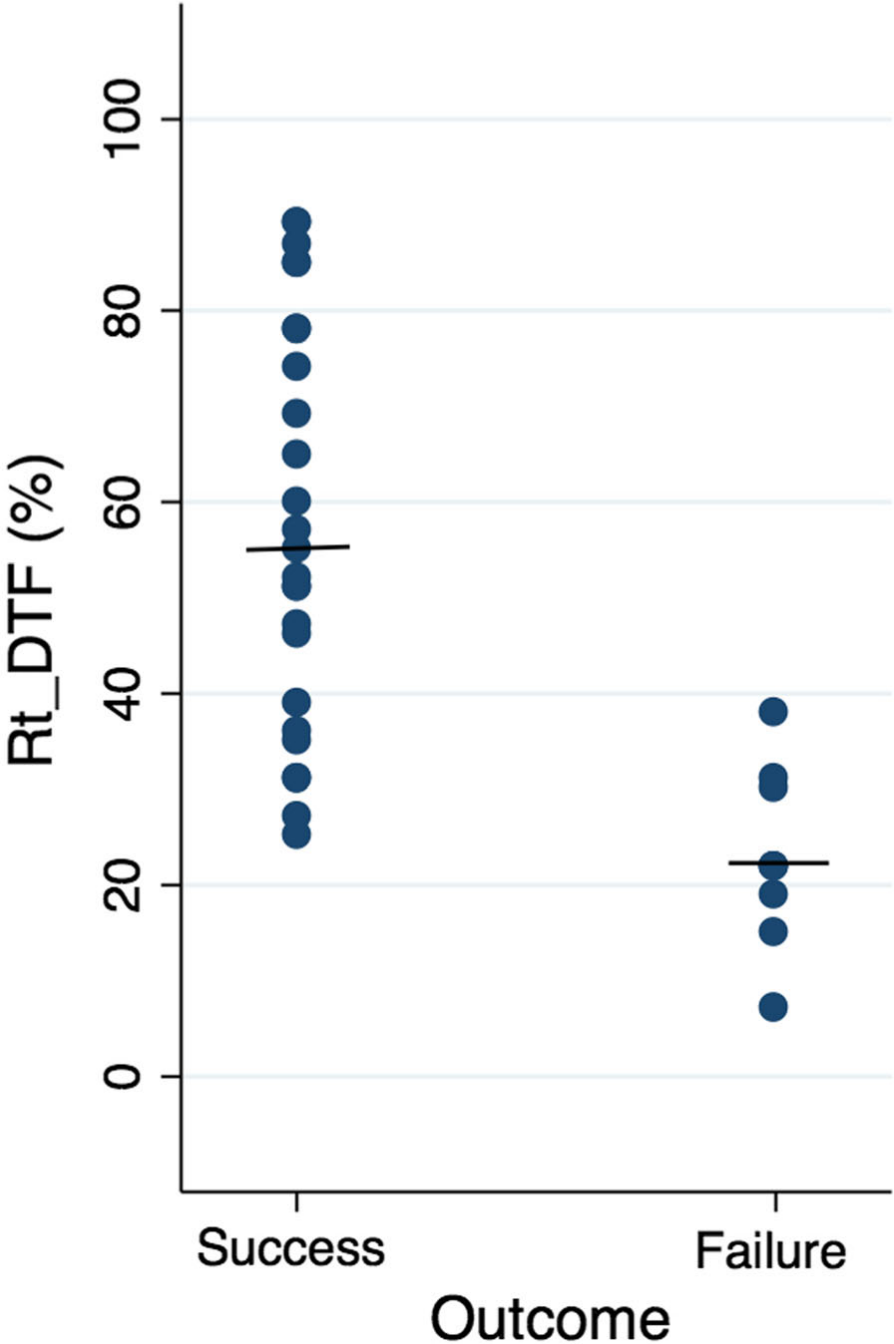
Dot plot of the right diaphragm thickening fraction in weaning success and failure groups (success group 57.7 ± 21.2%, failure group 22.9 ± 9.2%, *p* < 0.001)

Of the 34 patients enrolled, the mean age was 66.5 (± 13.5) years; 16 (47.1%) patients were male. All of the participants were ventilated through the endotracheal tube. Twenty-five patients (73.5%) were successfully weaned from the mechanical ventilator. The baseline characteristics were not significantly different between the weaning success and failure groups, as shown in Table 1. DTF in both sides were significantly different between patients who succeeded and failed SBT (right DTF: success group 57.7 ± 21.2%, failure group 22.9 ± 9.2%, *p* < 0.001 (Fig. 2); left DTF: success group 68.8 ± 41.2%, failure group 42.8 ± 18.5%, *p* = 0.017). There was a fair correlation between MIP and right DTF (*r* = 0.391, *p* = 0.022). In addition, other parameters displayed statistically significant differences between the success and failure groups: RSBI less than 105 (success group 96.0%, failure group 55.6%; *p* = 0.012) and MIP (success group − 33.8 ± 7.4 cmH_2_O, failure group − 27.8 ± 6.2 cmH_2_O; *p* = 0.037), shown in Table 2. In contrast, diaphragm thickness at TLC or RV alone did not have a statistically significant difference between groups, *p* = 0.450 and 0.433, respectively.

**Table 1:** Patient characteristics

Parameters (mean ± SD, otherwise unspecified)	All (*n* = 34)	Success (*n* = 25)	Failure (*n* = 9)	*p* value
Age (years)	66.5 ± 13.5	67.8 ± 13.0	62.7 ± 15.0	0.333
Male (%)	16 (47.1)	10 (40.0)	6 (66.7)	0.250
BMI (kg/m^2^)	22.3 ± 4.4	22.9 ± 4.1	20.6 ± 5.0	0.188
Indication for intubation (%)				
• Pneumonia	18 (52.9)	12 (48.0)	6 (66.7)	0.448
• Congestive heart failure/volume overload	8 (23.5)	7 (28.0)	1 (11.1)	0.403
• Sepsis other than pneumonia	4 (11.8)	3 (12.0)	1 (11.1)	1.000
• COPD/asthma	2 (5.9)	2 (8.0)	0 (0)	1.000
• Uremic encephalopathy	1 (2.9)	1 (4.0)	0 (0)	1.000
• TRALI	1 (2.9)	0 (0)	1 (11.1%)	0.265
Ventilator day (days)	10 ± 7.8	9.9 ± 7.4	12.3 ± 9.1	0.437
Previous SBT (%)	14 (41.2)	9 (36.0)	5 (55.6)	0.307
SpO_2_/F_I_O_2_ ratio	242.6 ± 4.5	242.6 ± 4.2	242.7 ± 5.7	0.988
Respiratory rate (/min)	18.9 ± 3.9	18.8 ± 4.1	19.1 ± 3.4	0.860
Minute ventilation (L/min)	7.3 ± 2.0	7.1 ± 2.0	7.9 ± 1.7	0.307

*SD* standard deviation, *BMI* body mass index, *COPD* chronic obstructive pulmonary disease, *TRALI* transfusion-related acute lung injury, *IQR* interquartile range, *SBT* spontaneous breathing trial, *SpO*_*2*_ blood oxygen saturation, *F*_*I*_*O*_*2*_ fraction of inspired oxygen

**Table 2:** Weaning parameters

Parameters (mean ± SD)	All (*n* = 34)	Success (*n* = 25)	Failure (*n* = 9)	*p* value
RSBI	52.6 ± 21.0	54.3 ± 22.8	47.7 ± 14.8	0.430
RSBI ≤ 105 (%)	29 (85.3)	24 (96.0)	5 (55.6)	0.012^a^
MIP (cmH_2_O)	− 32.2 ± 7.5	− 33.8 ± 7.4	− 27.8 ± 6.2	0.037^a^
MEP (cmH_2_O)	37.8 ± 14.8	38.4 ± 15.2	36.1 ± 14.5	0.698
Right diaphragm thickness at TLC (mm)	3.4 ± 1.3	3.5 ± 1.3	3.1 ± 1.3	0.450
Right diaphragm thickness at RV (mm)	2.4 ± 0.9	2.2 ± 0.9	2.5 ± 1.1	0.433
Right DTF (%)	48.5 ± 24.3	57.7 ± 21.2	22.9 ± 9.2	< 0.001^a^
Left DTF (%)	61.9 ± 38.1	68.8 ± 41.2	42.8 ± 18.5	0.017^a^

*RSBI* rapid shallow breathing index, *SD* standard deviation, *MIP* maximum inspiratory pressure, *MEP* maximum expiratory pressure, *TLC* total lung capacity, *RV* residual volume, *DTF* diaphragm thickening fraction

^a^Statistically significant

**Fig. 3:**
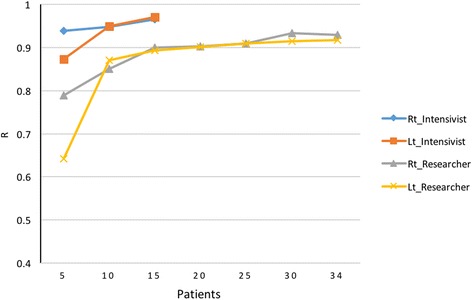
Intra-observer correlation of the researcher (SR) and intensivist (PP)

Inter-observer correlation was significantly high in the first 15 patients on both sides (right diaphragm, *r* = 0.89, *p* < 0.001; left diaphragm, *r* = 0.89, *p* < 0.001). Intra-observer correlation of the co-author (SR) (Fig. 3) increased sharply to almost 0.9 in the first ten patients and slightly increased after that. There were highly significant intra-observer correlations of both diaphragms in the all patients (*r* = 0.93, *p* < 0.001 for right diaphragm; *r* = 0.92, *p* < 0.001 for left diaphragm).

**Fig. 4:**
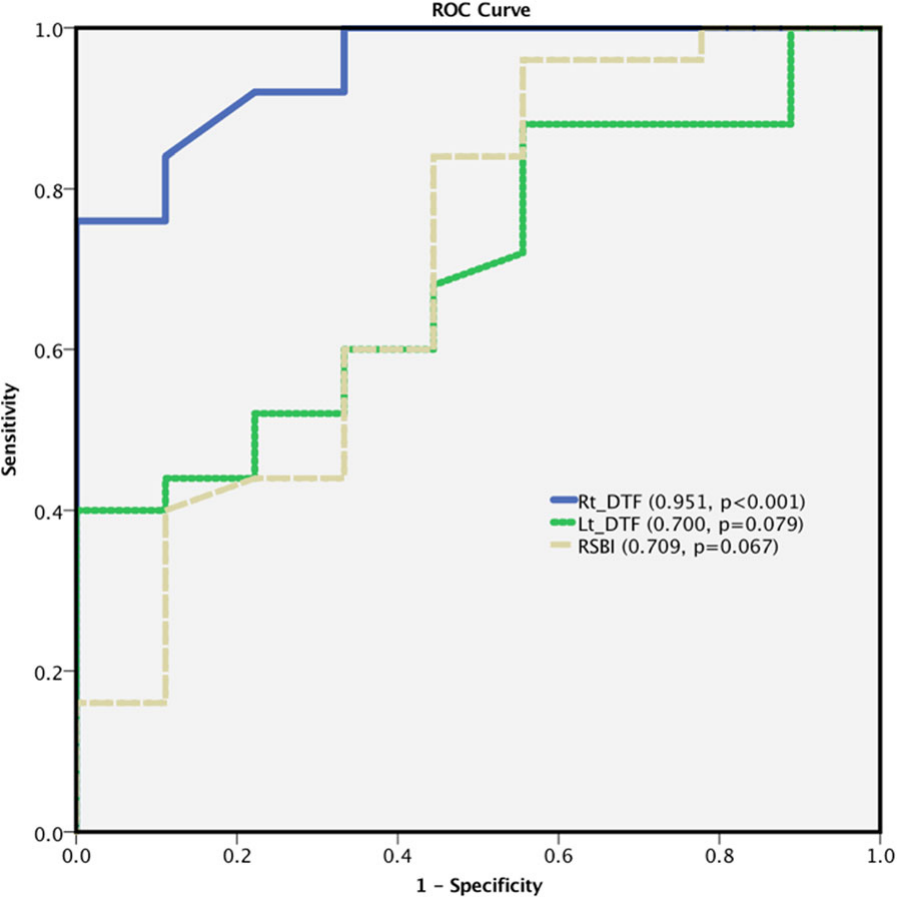
Receiver operating characteristic curve for right DTF: AUC 0.951 (95% CI 0.88–1.00; *p* < 0.001), left DTF: AUC 0.709 (95% CI 0.50–0.92; *p* = 0.067), and RSBI: AUC 0.700 (95% CI 0.52–0.88; *p* = 0.079)

The ROC curves of right and left DTF and the RSBI for the prediction of successful weaning were 0.951, 0.700, and 0.709, respectively (Fig. 4). The best cutoff value for predicting weaning successfulness was right DTF of more than or equal to 26%, which had the highest accuracy of 88.2%, sensitivity of 96.0%, specificity of 67.7%, PPV of 88.9%, and NPV of 85.7% (Table 3). A combination of right DTF of more than or equal to 26% and RSBI less than or equal to 105 had an accuracy of 88.2%, sensitivity of 92.0%, specificity of 77.8%, PPV of 92.0%, and NPV of 77.8%.

**Table 3:** Comparison between diagnostic tests

Diagnostic test	Sensitivity (%)	Specificity (%)	PPV (%)	NPV (%)	Accuracy (%)
RSBI ≤ 105	96.0	44.4	82.8	80.0	82.4
Right DTF ≥ 26%	96.0	67.7	88.9	85.7	88.2
Right DTF ≥ 26% and RSBI ≤ 105	92.0	77.8	92.0	77.8	88.2

*RSBI* rapid shallow breathing index, *DTF* diaphragm thickening fraction, *PPV* positive predictive value, *NPV* negative predictive value

## Discussion

Our study found that a right DTF of more than or equal to 26% had the most accuracy for predicting weaning successfulness. This result is consistent with the studies from Ferrari et al. [22], DiNino et al. [27], Dube et al. [18], and Blumhof et al. [28] which demonstrated that right DTFs of more than 36, 30, 29, and 20%, respectively, were associated with weaning success and better ICU outcomes. Most of these studies confirmed DTF alone as a better weaning predictor compared to RSBI, in terms of specificity without sensitivity extenuation. From our data, the combination of right DTF of more than or equal to 26% and RSBI less than or equal to 105 increased specificity for predicting the successfulness of SBT compared to RSBI alone. This helps the physician wean critically ill patients more confidently. However, the accuracy of the above combination was not different from using DTF alone; this finding duplicates a previous study [28]. In the future, DTF might replace RSBI as a weaning parameter.

Patients who cannot tolerate independent breathing tend to breathe rapidly (high respiratory rate) and shallowly (low tidal volume) [29], which could reflect diaphragm weakness. RSBI, introduced in 1991 [8], had a high area under the ROC curve of 0.89 compared to 0.709 in our study. The lesser accuracy of RSBI for us in predicting weaning successfulness could possibly be due to population diversity as ours was older (66.5 ± 13.5 vs 59.6 ± 1.7 years of age) and perhaps a different ethnic group. Because respiratory function declines gradually over a lifetime [30, 31], the accuracy of RSBI might concurrently decrease with increased patient age. Measuring diaphragmatic function by DTF predicted the successfulness of SBT, with the right DTF being more accurate than the left DTF. The reason for the improved ability in determining weaning success using the right could be from larger lung volume (ten subsegments compared to eight subsegments on the left) [32]. MIP could also distinguish the weaning success and failure groups and emphasized the importance of diaphragmatic function. Nevertheless, the absolute number of diaphragmatic thickness at TLC or RV did not show statistically significant differences between the two weaning groups, similarly reported in the previous study [18].

Comparing diaphragm thickness in our study with the previous one from Ferrari et al. [22], patients with weaning success had a diaphragm thickness at TLC of 3.5 ± 1.3 and 3.8 mm (IQR 2.9–4.5), respectively, and at RV of 2.2 ± 0.9 mm and 2.5 mm (IQR 1.9–2.8), respectively. Patients with weaning failure had a diaphragm thickness at TLC of 3.1 ± 1.3 mm and 3.0 mm (IQR 2.0–4.0), respectively, and at RV of 2.5 ± 1.1 mm and 2.4 mm (IQR 1.7–3.0), respectively. The right DTF of the weaning success group for us was 57.7 ± 21.2, almost the same as in the previous study of 56 (IQR 38–64) [22], while in the weaning failure group, the right DTF in our study was 22.9 ± 9.2, slightly lower than that from the previous study of 26 (IQR 22–30) [22]. The similarity of these two studies highlights the reproducibility of diaphragm thickness and DTF [26]; effects from the type of artificial airways in terms of endotracheal tube (all of our patients) or tracheostomy tube (all patients in the previous study) are negligible on the results.

As ultrasound is an operator-dependent method, some important techniques were used to decrease inter-observer variation. First, the patients’ posture was identical and anatomical landmarks were applied for positioning the probe. Second, measurement of the diaphragm thickness at TLC and RV was conducted intra-breath to decrease effort error between breath. Because of that, we found a highly noticeable inter-observer correlation on both sides of the diaphragm (right diaphragm, *r* = 0.89, *p* < 0.001; left diaphragm, *r* = 0.89, *p* < 0.001).

Limitations include the relatively small number of patients and single-center enrollment. Nevertheless, our results were in accordance with the previously mentioned research and acknowledged the reproducibility of DTF even in a resource-limited setting as ours. Moreover, one of the researchers (SR) is a pulmonology fellow and not an intensivist, with some experience on thoracic ultrasound. However, the excellent learning curve in measuring DTF can be certainly achieved, as demonstrated from our intra-observer correlation which increased sharply to almost 0.9 in the first ten patients. This also emphasizes the usefulness of DTF in a general setting: a well-trained point-of-care ultrasound intensivist is not necessary. One of the strengths in our study was our collection of data from the left DTF, not presented in other research; it was significantly different between our two groups but not with as much variation as with the right.

## Conclusions

Diaphragm thickening fraction of the right diaphragm by ultrasound of more than or equal to 26% combined with RSBI of less than or equal to 105 improved the efficacy for prediction of successful weaning compared to RSBI alone. Point-of-care ultrasound to assess diaphragm function has a steep learning curve but is definitely achievable and with excellent reproducibility. This combination could help physicians assess weaning readiness in critically ill patients, relatively easy to manage and cost effective.

## Abbreviations

DTF: Diaphragmatic thickening fraction
F_I_O_2_: Fraction of inspired oxygen
IQR: Interquartile range
MEP: Maximum expiratory pressure
MIP: Maximum inspiratory pressure
NPV: Negative predictive value
P 0.1: Tracheal airway occlusion pressure 0.1 s
PaO_2_: Partial pressure of oxygen in arterial blood
PEEP: Positive end-expiratory pressure
PPV: Positive predictive value
ROC: Receiver operating characteristic
RSBI: Rapid shallow breathing index
RV: Residual volume
SBT: Spontaneous breathing trial
SD: Standard deviation
SpO_2_: Pulse oxygen saturation
TLC: Total lung capacity

## References

[1] Vivier E, Mekontso Dessap A, Dimassi S, Vargas F, Lyazidi A, Thille AW (2012). Diaphragm ultrasonography to estimate the work of breathing during non-invasive ventilation. Intensive Care Med.

[2] Wait JL, Nahormek PA, Yost WT, Rochester DP (1989). Diaphragmatic thickness-lung volume relationship in vivo. J Appl Physiol.

[3] Boles JM, Bion J, Connors A, Herridge M, Marsh B, Melot C (2007). Weaning from mechanical ventilation. Eur Respir J.

[4] Esteban A, Frutos F, Tobin MJ, Alia I, Solsona JF, Valverdu I, et al. A comparison of four methods of weaning patients from mechanical ventilation. Spanish Lung Failure Collaborative Group. N Engl J Med 1995; 332:345-50. 10.1056/NEJM199502093320601 7823995

[5] Mancebo J (1996). Weaning from mechanical ventilation. Eur Respir J.

[6] Cook DJ, Walter SD, Cook RJ, Griffith LE, Guyatt GH, Leasa D (1998). Incidence of and risk factors for ventilator-associated pneumonia in critically ill patients. Ann Intern Med.

[7] Blackwood B, Alderdice F, Burns K, Cardwell C, Lavery G, O’Halloran P (2011). Use of weaning protocols for reducing duration of mechanical ventilation in critically ill adult patients: Cochrane systematic review and meta-analysis. BMJ.

[8] Yang KL, Tobin MJ (1991). A prospective study of indexes predicting the outcome of trials of weaning from mechanical ventilation. N Engl J Med.

[9] Bien Udos S, Souza GF, Campos ES, Farah de Carvalho E, Fernandes MG, Santoro I (2015). Maximum inspiratory pressure and rapid shallow breathing index as predictors of successful ventilator weaning. J Phys Ther Sci.

[10] Okamoto K, Sato T, Morioka T (1990). Airway occlusion pressure (P0.1)—a useful predictor for the weaning outcome in patients with acute respiratory failure. J Anesth.

[11] El-Khatib MF, Bou-Khalil P (2008). Clinical review: liberation from mechanical ventilation. Crit Care.

[12] Petrof BJ, Hussain SN (2016). Ventilator-induced diaphragmatic dysfunction: what have we learned?. Curr Opin Crit Care.

[13] Petrof BJ, Jaber S, Matecki S (2010). Ventilator-induced diaphragmatic dysfunction. Curr Opin Crit Care.

[14] Daniel Martin A, Smith BK, Gabrielli A (2013). Mechanical ventilation, diaphragm weakness and weaning: a rehabilitation perspective. Respir Physiol Neurobiol.

[15] Goligher EC, Fan E, Herridge MS, Murray A, Vorona S, Brace D (2015). Evolution of diaphragm thickness during mechanical ventilation. Impact of inspiratory effort. Am J Respir Crit Care Med.

[16] Nason LK, Walker CM, McNeeley MF, Burivong W, Fligner CL, Godwin JD (2012). Imaging of the diaphragm: anatomy and function. Radiographics.

[17] Johnson NE, Utz M, Patrick E, Rheinwald N, Downs M, Dilek N (2014). Visualization of the diaphragm muscle with ultrasound improves diagnostic accuracy of phrenic nerve conduction studies. Muscle Nerve.

[18] Dube BP, Dres M, Mayaux J, Demiri S, Similowski T, Demoule A (2017). Ultrasound evaluation of diaphragm function in mechanically ventilated patients: comparison to phrenic stimulation and prognostic implications. Thorax.

[19] Kharma N (2013). Dysfunction of the diaphragm: imaging as a diagnostic tool. Curr Opin Pulm Med.

[20] Laporta D, Grassino A (1985). Assessment of transdiaphragmatic pressure in humans. J Appl Physiol.

[21] Vassilakopoulos T, Zakynthinos S, Roussos C (1998). The tension-time index and the frequency/tidal volume ratio are the major pathophysiologic determinants of weaning failure and success. Am J Respir Crit Care Med.

[22] Ferrari G, De Filippi G, Elia F, Panero F, Volpicelli G, Apra F (2014). Diaphragm ultrasound as a new index of discontinuation from mechanical ventilation. Crit Ultrasound J.

[23] Lerolle N, Guerot E, Dimassi S, Zegdi R, Faisy C, Fagon JY (2009). Ultrasonographic diagnostic criterion for severe diaphragmatic dysfunction after cardiac surgery. Chest.

[24] Boussuges A, Gole Y, Blanc P (2009). Diaphragmatic motion studied by m-mode ultrasonography: methods, reproducibility, and normal values. Chest.

[25] MacIntyre NR, Cook DJ, Ely EW, Epstein SK, Fink JB, Heffner JE (2001). Evidence-based guidelines for weaning and discontinuing ventilatory support: a collective task force facilitated by the American College of Chest Physicians; the American Association for Respiratory Care; and the American College of Critical Care Medicine. Chest.

[26] Ueki J, De Bruin PF, Pride NB (1995). In vivo assessment of diaphragm contraction by ultrasound in normal subjects. Thorax.

[27] DiNino E, Gartman EJ, Sethi JM, McCool FD (2014). Diaphragm ultrasound as a predictor of successful extubation from mechanical ventilation. Thorax.

[28] Blumhof S, Wheeler D, Thomas K, McCool FD, Mora J (2016). Change in diaphragmatic thickness during the respiratory cycle predicts extubation success at various levels of pressure support ventilation. Lung.

[29] Tobin MJ, Perez W, Guenther SM, Semmes BJ, Mador MJ, Allen SJ (1986). The pattern of breathing during successful and unsuccessful trials of weaning from mechanical ventilation. Am Rev Respir Dis.

[30] Fletcher C, Peto R (1977). The natural history of chronic airflow obstruction. Br Med J.

[31] Hautmann H, Hefele S, Schotten K, Huber RM (2000). Maximal inspiratory mouth pressures (PIMAX) in healthy subjects—what is the lower limit of normal?. Respir Med.

[32] Ugalde P, Camargo Jde J, Deslauriers J (2007). Lobes, fissures, and bronchopulmonary segments. Thorac Surg Clin.

