# Correction to: Use of diaphragm thickening fraction combined with rapid shallow breathing index for predicting success of weaning from mechanical ventilator in medical patients

**DOI:** 10.1186/s40560-018-0293-9

**Published:** 2018-04-23

**Authors:** Pattarin Pirompanich, Sasithon Romsaiyut

**Affiliations:** Division of Pulmonary and Critical Care Medicine, Department of Medicine, Faculty of Medicine, Thammasat University, Pathumthani, Thailand

## Correction

The original article [1] contained an error. The caption of Fig. 4 should be changed. The correct caption can be found below.

“Fig. 4 Receiver operating characteristic curve for right DTF: AUC 0.951 (95% CI 0.88–1.00; p < 0.001), left DTF: AUC 0.700 (95% CI 0.52–0.88; p = 0.079), and RSBI: AUC 0.709 (95% CI 0.50–0.92; p = 0.067)”.
